# Factors affecting anxiety in patients with multiple sclerosis

**DOI:** 10.1192/j.eurpsy.2025.641

**Published:** 2025-08-26

**Authors:** N. Ben Hamed, K. Mahfoudh, A. Aissa, A. Souissi, R. Jomli

**Affiliations:** 1 psychiatry A. razi hospital; 2 neurology department. LR18SP03 and Clinical Investigation Center Neurosciences and Mental Health - university hospital center Razi, Manouba, Tunisia

## Abstract

**Introduction:**

Anxiety is a common and often debilitating condition in individuals with Multiple Sclerosis (MS), significantly affecting their quality of life. The challenges associated with managing MS symptoms and the potential for disability can contribute to increased levels of psychological distress.

**Objectives:**

The aim of our study was to determine the prevalence of anxiety and identify the its associated factors.

**Methods:**

A cross-sectional study was conducted in the neurology department of Razi University Hospital (Tunisia) between October 2023 and June 2024. Patients with a diagnosis of MS based on the 2017 McDonald criteria were recruited, excluding those with active disease relapses. Participants completed questionnaires covering sociodemographic data, medical history, clinical and radiological characteristics, disability status, and psychological symptoms. Depression, anxiety and stress were assessed using the DASS-21 scale. Insomnia was evaluated using the Pittsburgh Sleep Quality Index (PSQI). Data analysis was performed using SPSS version 26.

**Results:**

A total of 83 patients with MS were recruited, with ages ranging from 19 to 66 years. The study population had a predominantly female sex ratio of 3.4. The majority of participants (75.9%) were from urban areas, and 74.7% had a university-level education. Moreover, 49.1% were married, and 60.2% were employed. Regarding medical history, 40.3% had a comorbid condition, and 30.1% had a psychiatric history. The mean age at disease onset was 26 ± 10 years, and the most common clinical presentations were sensory and pyramidal symptoms.

The median time since the last relapse in our sample was 24 months. In our sample, first-line treatments (interferon, glatiramer acetate, teriflunomide, dimethyl fumarate) were prescribed to 27.7% of patients. Second-line treatments (natalizumab, ocrelizumab, fingolimod) were prescribed to 69.9% of patients

In our study, the prevalence of anxiety was 55.4%. In our population. 26.5% of the patients had severe anxiety. A significant association was found between anxiety and female gender (p=0.02), stress (p<0.001), and insomnia (p=0.003).

**Conclusions:**

The findings indicate that anxiety is a considerable concern for individuals with MS. Addressing this mental health issue is essential for healthcare providers to offer effective support. By prioritizing mental health, we can enhance the overall well-being of individuals living with MS and improve their quality of life.

**Disclosure of Interest:**

None Declared

